# High prevalence of parasitic chytrids infection of glacier algae in cryoconite holes in Alaska

**DOI:** 10.1038/s41598-023-30721-w

**Published:** 2023-03-09

**Authors:** Kino Kobayashi, Nozomu Takeuchi, Maiko Kagami

**Affiliations:** 1 Graduate School of Science and Engineering, Chiba University, Chiba, Japan; 2 Department of Earth Sciences, Graduate School of Science, Chiba University, Chiba, Japan; 3 Faculty of Environment and Information Sciences, Yokohama National University, Kanagawa, Japan

**Keywords:** Microbiology, Environmental sciences

## Abstract

Glacier algae, which are photosynthetic microbes growing on ice, considerably reduce the surface albedo of glaciers and accelerate their melting rate. Although the growth of glacier algae can be suppressed by parasitic chytrids, the impact of chytrids on algal populations is still largely unknown. In this study, we described the morphology of the chytrid infecting the glacier alga *Ancylonema nordenskioeldii* and quantified the prevalence of infection in different habitats on a mountain glacier in Alaska, USA. Microscopic observations revealed three different morphological types of chytrids with distinct rhizoid shapes. Variations in the size of the sporangia were probably because of their different growth stages, indicating that they actively propagated on the glacier. The prevalence of infection did not vary among sites with different elevations but was substantially higher in cryoconite holes (20%) than on ice surfaces (4%) at all sites. This indicates that cryoconite holes are hot spots for chytrid infections of glacier algae, and the dynamics of cryoconite holes might affect the host–parasite interactions between chytrids and the glacier algae, which may in turn alter surface albedo and ice melting.

## Introduction

Melting glaciers and snowpacks in polar and high-mountain regions are ecosystems comprising various living organisms. Diverse photosynthetic microbes including algae and cyanobacteria, grow on snow and ice surfaces^1^, sustaining the heterotrophic organisms, including insects such as Plecoptera and Collembola^2,3^ and micro-invertebrates such as tardigrades and rotifers^4,5^, inhabiting these regions. Diverse bacteria and fungi are also active on glaciers and snowpacks worldwide, as revealed by microscopic observations, DNA analyses, and cultivation^6–8^. Associations among such organisms across glacial and snowpack habitats are important to assess the biogeochemical cycles and population dynamics of each species in the community structure.

Chytrids are true fungi that form free-swimming zoospores^9,10^. They include saprophytic and parasitic species^11^. Parasitic chytrids infect various organisms, including vertebrates, invertebrates, vascular plants, and algae, leading to their death^11^. Chytrids are widely distributed in aquatic and terrestrial environments^11^ and have a great influence on ecosystems because their infections can largely control host population dynamics^12^. For example, chytrids parasitizing some frogs have caused their extinction in Central America and Australia^13^. Chytrids parasitizing phytoplankton in lakes can drastically reduce the host population and/or terminate blooms, and are thus strong drivers of the seasonal succession of the phytoplankton community^12^.

Chytrids have recently been reported in cold environments such as polar oceans, alpine soil, snowpacks, and glaciers. For example, chytrids parasitizing diatoms are widely distributed in the Arctic ocean^14^ and their infectivity changes with the salinity and solar irradiance of seawater, particularly as a result of sea-ice melting^15^. Furthermore, chytrids were found in glacial snow and ice as well as in naked soil in moraines in South America via DNA analyses^16^. In alpine soil, chytrids are dominant owing to the water-logged conditions under the snow^17^. Environmental DNA analyses of snowpacks in North America and Europe revealed the presence of a novel clade of chytrids referred to as the ‘Snow Clade,’ which consisted only of sequences from cold environments, suggesting their long history of evolution in the cryosphere^18^.

Parasitic chytrids on glacier algae were first reported by Kol^19^ from the surface of the Colombia Glacier in Alaska. The author described a chytrid infecting *Ancylonema nordenskioeldii* and named it *Rhizophydium sphaerocarpum* based on the morphology of the sporangia and rhizoid. On Svalbard glaciers, chytrids parasitizing *A. nordenskioeldii* and the snow alga, *Sanguina nivaloides*, have also been found^20^. Chytrids were also observed in incubation experiments with glacier algae collected from the Greenland Ice Sheet^7^. However, no quantitative analysis has yet been conducted; thus, the impacts of chytrids on glacier and snow algae is still unknown.

Can chytrids greatly impact glacier ecosystems by controlling the population dynamics of glacier algae? Glacier algae, which are a group of surface ice-inhabiting Streptophytes^21^, significantly affect the surface albedo of glaciers and accelerate the melting rate of ice surfaces because of the dark-colored pigments present in their vacuoles^21–23^, which change the color of the ice surface to purple or brown during a bloom^21,22,24–26^. The colored ice surface absorbs more solar radiation than the original white ice surface without algae, consequently accelerating the ice-melting rate^27,28^. If chytrid infections control the population of glacier algae on ice surfaces, the spread of chytrid infection may reduce ice darkening by cryoflora and slow down glacier melting. Therefore, it is important to investigate the dynamics of chytrids in relation to glacier algae.

Microbial activity varies with habitats arising from the heterogeneous topography of glaciers^29^. Differences in hydrological conditions among habitats may affect the distributions of chytrids because chytrid zoospores have to swim to actively find a host. One of the main glacial habitats is the ice surface, which usually has a porous structure in the surface layer (weathering crust) wetted by meltwater. Glacier algae preferably grow in gaps in the porous ice^30^, which may enhance chytrid infection. Another habitat is cryoconite holes, which are small, cylindrical, water-filled pits formed on ice surfaces^31,32^. The water-rich cryoconite holes have sediments of the cryoconite formed by filamentous cyanobacteria, and host diverse and abundant microbes, including algae, bacteria, fungi, and micro-invertebrates^4,33–35^, with chytrids also possibly growing in them. Various factors are known to influence alga–chytrid interactions^36^, and water turbulence is one factor preventing chytrid infections^37^. Thus, the infection rate may change with the dimensions and hydrology of the cryoconite holes, which are usually determined by weather conditions^38^.

**Figure 1:**
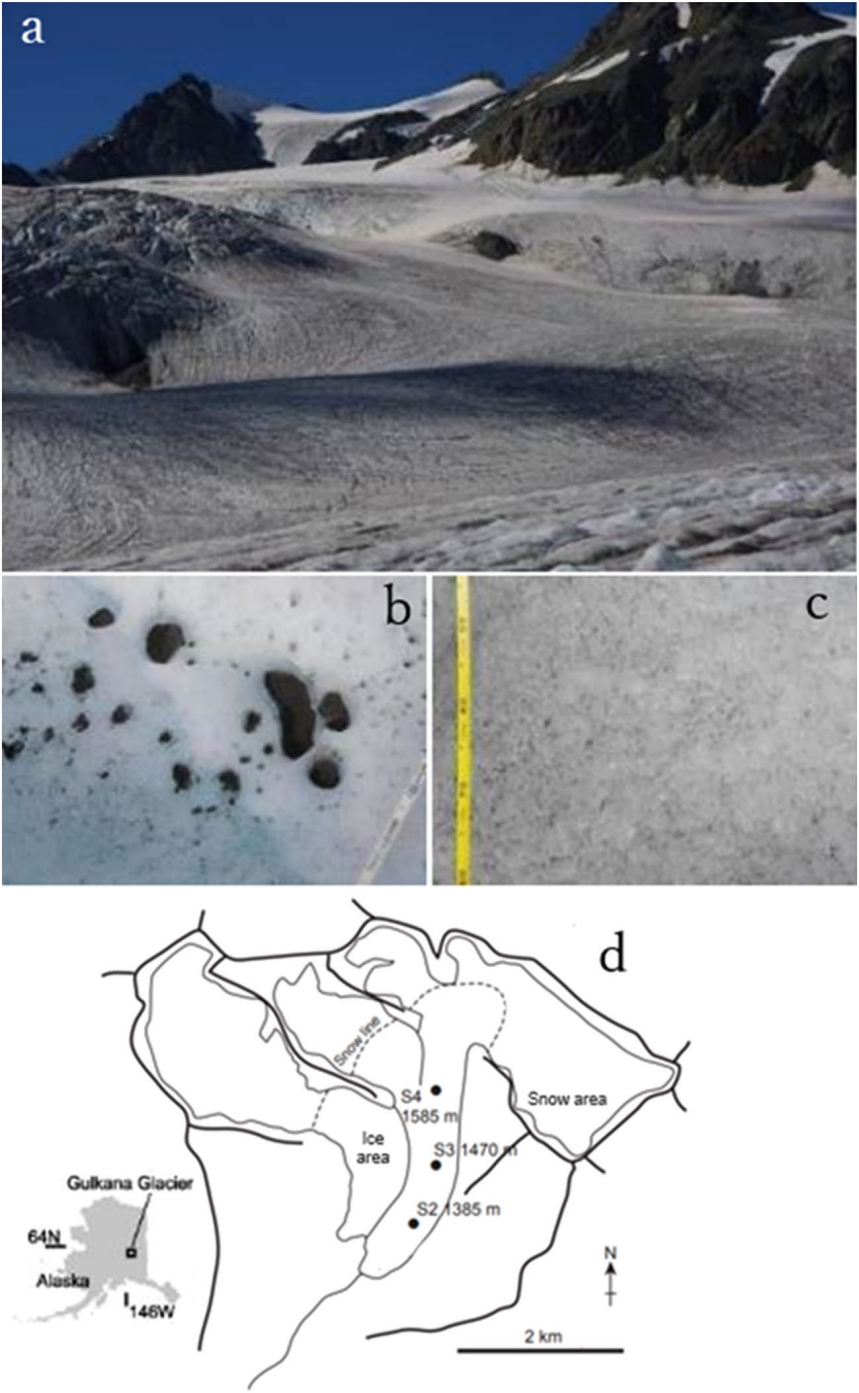
The study site, Gulkana Glacier in Alaska, USA. Photographs of the area around site S4 on the glacier taken on 6 August, 2015 (**a**), cryoconite holes (**b**), and ice surfaces (**c**). A map of the glacier showing sampling sites generated using illustrator software (**d**).

This study aimed to describe the morphology of the chytrids infecting glacier algae and examine their distribution quantitatively in the bare-ice area of the Gulkana Glacier in Alaska, USA (Fig. 1), where diverse microbes have been reported^1,39,40^. The chytrids was not only quantified but also somewhat classified. The prevalence of chytrid infection in algal cells were quantified at different elevations and habitats on the glacier to understand the impact and dynamics of parasitic chytrids on glacier algae.

## Results

### Microscopy of glacier algae and chytrids

**Figure 2:**
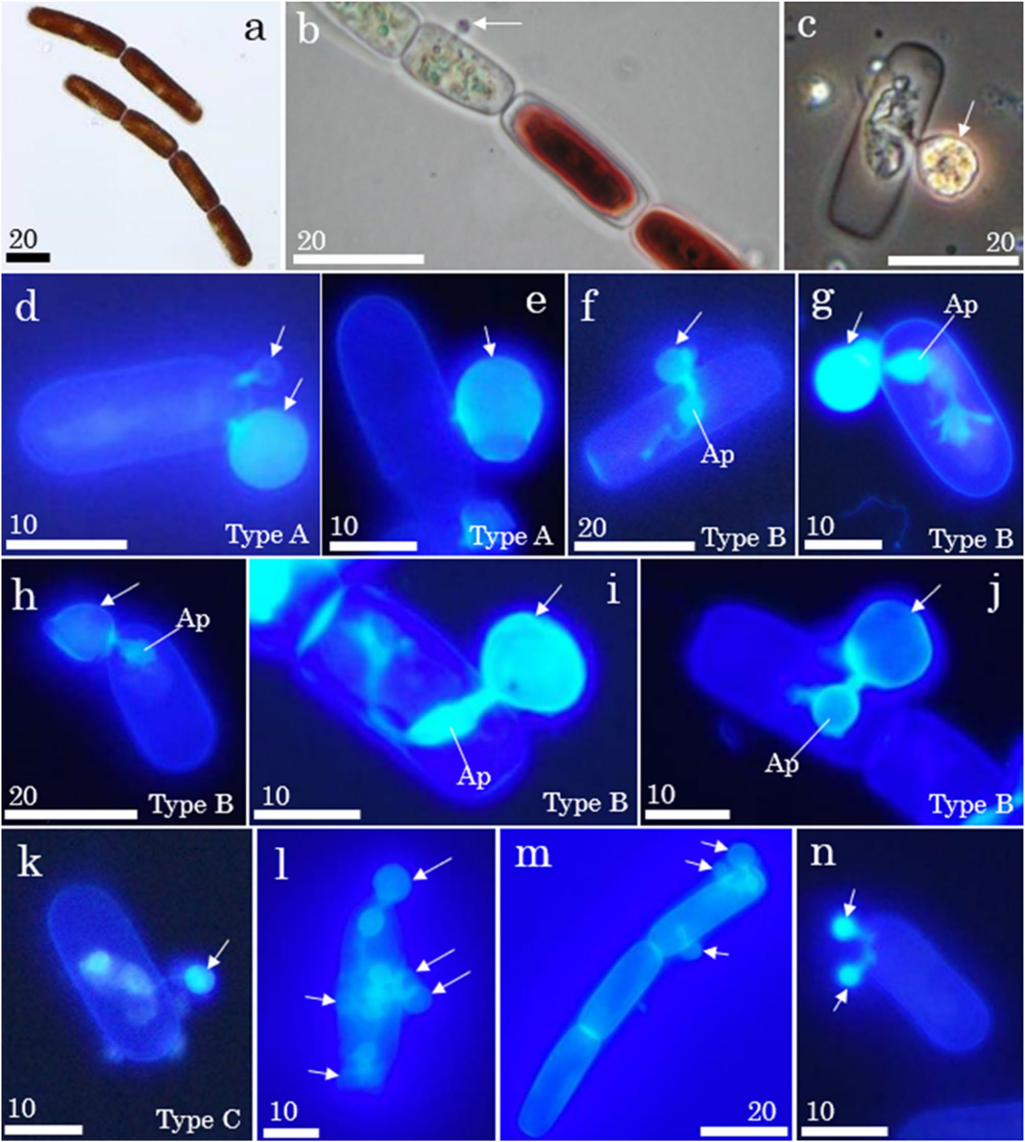
Brightfield (**a**–**c**) and fluorescence (**d**–**n**) microscope photographs of the glacier algae, *Ancylonema nordenskioeldii* (**a**), and those infected with chytrids (**b**,**c**). The algal cells infected with chytrids lost their dark-colored pigment (**b**). Type A chytrids with a short rhizoid (**d**,**e**). Type B chytrids with a long rhizoid with an apophysis (**f**–**j**). Two subtypes of Type B chytrids: the elliptical apophysis with the rhizoid extended in a straight line from the sporangium (**i**); the spherical apophysis with the branched rhizoid extended from the sporangium in a direction other than a straight line (**j**). Type C chytrids with the rhizoid extending outside the cell but no rhizoids inside the host cell (**k**). Algal cells infected with multiple chytrids (**l**–**n**). The chytrids are marked with arrows. Apophysis (Ap). All scale bars are in micrometers.

**Figure 3:**
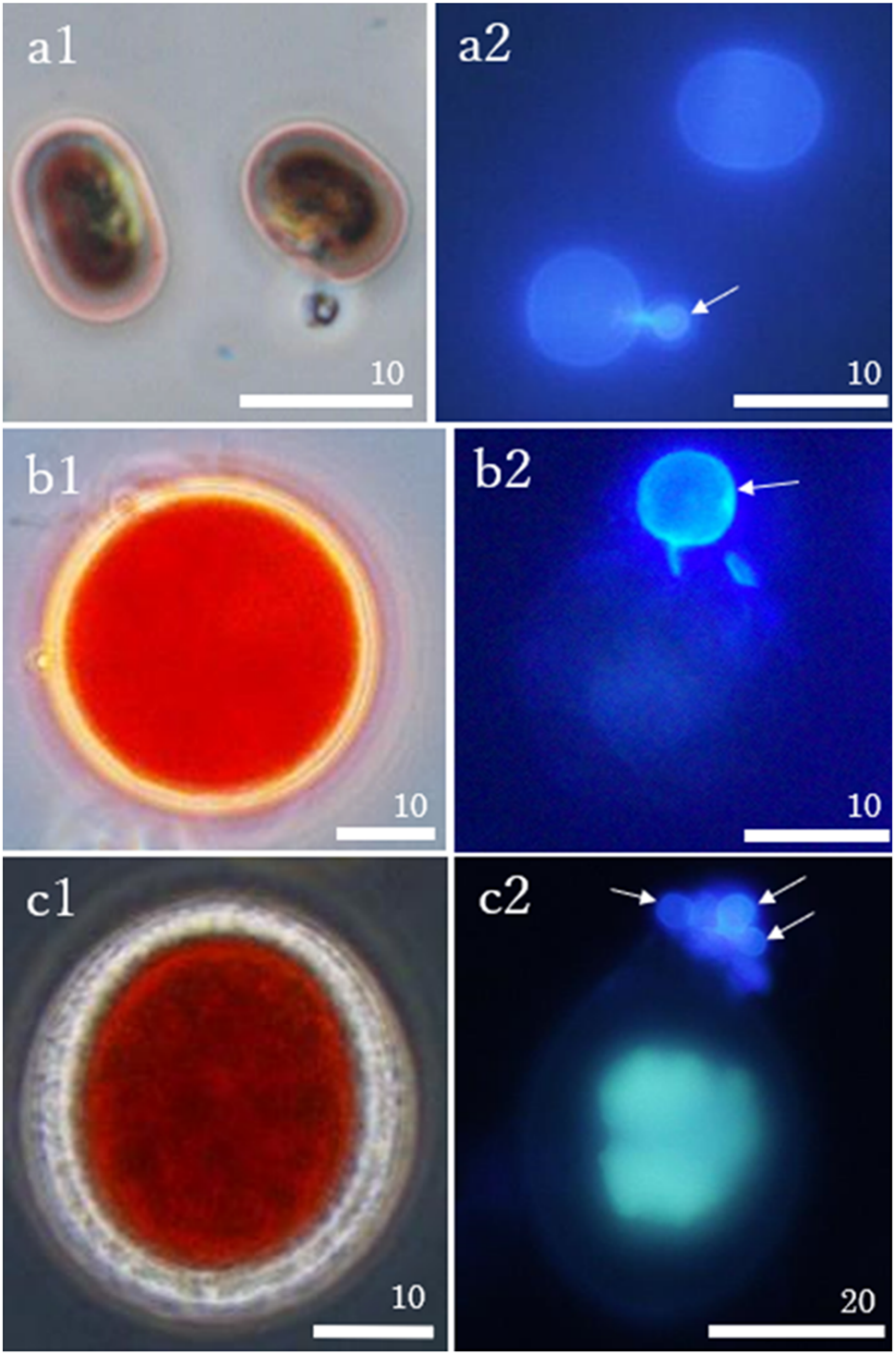
Microscopic photographs of cryoflora from Gulkana Glacier. *Ancylonema alaskana* (**a1**), the snow algae *Sanguina nivaloides* (**b1**) and *Chloromonas* sp. (**c1**), and those infected with chytrids (**a2**,**b2**,**c2**). The chytrids are marked with arrows. All scale bars are in micrometers.

The glacier alga *A. nordenskioeldii* (Fig. 2a,b) was abundant in all samples analyzed in this study. The cell concentration ranged from 3.9 × 10^3^ to 3.4 × 10^5^ cells mL^−1^. The algae were 27.0 ± 7.3 μm long and 10.6 ± 1.0 μm wide (mean ± standard deviation). The algae were often connected and formed filaments consisting of 1–16 cells (Fig. 2a). Another glacier algae, *A. alaskana* (renamed from *Mesotaenium bergrenii*^41^), was also observed in all samples. Snow algae *S. nivaloides*. (renamed from *Chlamydomonas nivalis*^42^) and *Chloromonas* sp. (based on the molecular data of snow algae on this glacier^43^) were also observed in samples from site S4. All these algal taxa were observed to be infected by chytrids (Fig. 3a–c).

Three main morphological types of chytrids were observed based on light microscopy of their rhizoids. Type A chytrids had a relatively short rhizoid that extended into the host cells. The rhizoid was shorter than or equivalent to the diameter of the sporangium (Fig. 2d,e). The sporangia were spherical and some lacked an operculum (Fig. 2e). Type B chytrids had a long rhizoid that extended into the host cells. The length of the rhizoid exceeded the sporangium diameter. Most of the rhizoids had a bulge called a sub-sporangial apophysis. The size of the apophysis was smaller than or equivalent to that of the sporangium (Fig. 2f–j). In some cases, the rhizoid extended further from the apophysis and diverged multiple times. The shape of the apophysis was either elliptical with an extended rhizoid (Fig. 2i) or spherical with less rhizoid extension (Fig. 2j). The sporangia were lacking an operculum (Fig. 2h). Type C chytrids had no rhizoids inside the host cell but had one extending outside the cell. No apophysis or branches were found in the rhizoids (Fig. 2k).

**Figure 4:**
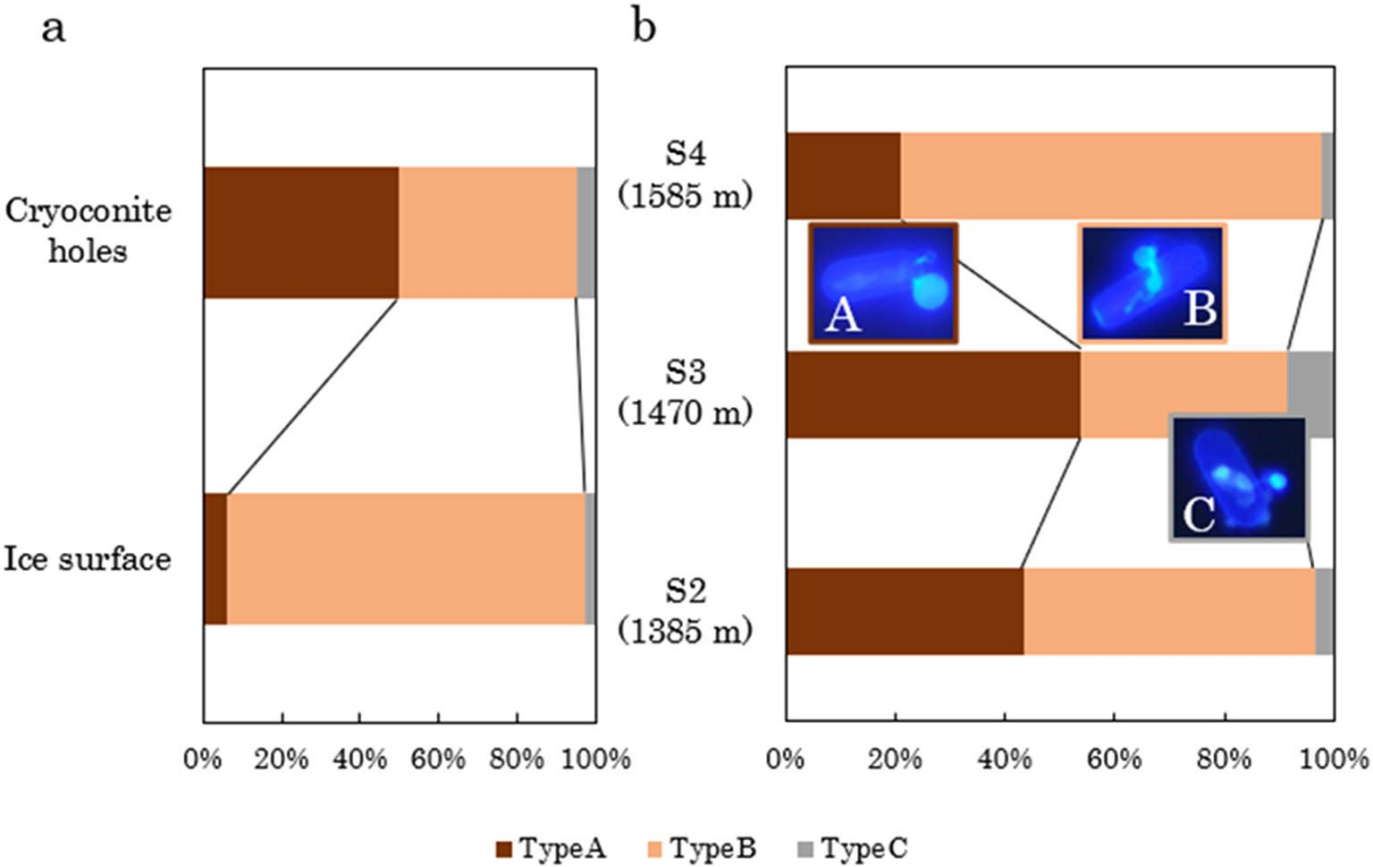
Proportion of the abundance of the three morphological types of chytrids (Types A, B, and C) in two different habitats (cryoconite holes and ice surfaces) (**a**) and at the three study sites (S2, S3, and S4) (**b**).

Types A, B, and C chytrids accounted for 39.6%, 55.9%, and 4.5% of the total chytrids (n = 288), respectively. Type B chytrids were most dominant on the ice surface (91.0%), whereas Type A and B chytrids were equally dominant (49.8% and 45.0%) in the cryoconite holes (Fig. 4). Type B chytrids were also dominant at the most elevated site, S4 (76.8%), whereas Type A and B chytrids were equally dominant at the lower elevation sites, S2 and S3.

### Prevalence of chytrid infection in algal cells

The prevalence of chytrid infection in the cells of *A. nordenskioeldii* ranged from 0% to 32.1% (mean: 12.3 ± 10.6%) on this glacier. Most infected cells had a single chytrid spore (Fig. 2c,e–k), although some had multiple spores (Fig. 2l–n). The maximum number of chytrid spores infecting a single algal cell (i.e., the number of chytrid cells per single algal cell) was five (Fig. 2l). The sizes of the chytrid’s sporangia ranged from 0.98 to 11.9 μm (mean: 4.6 μm, Supplementary Fig. S1, Table S1).

**Figure 5:**
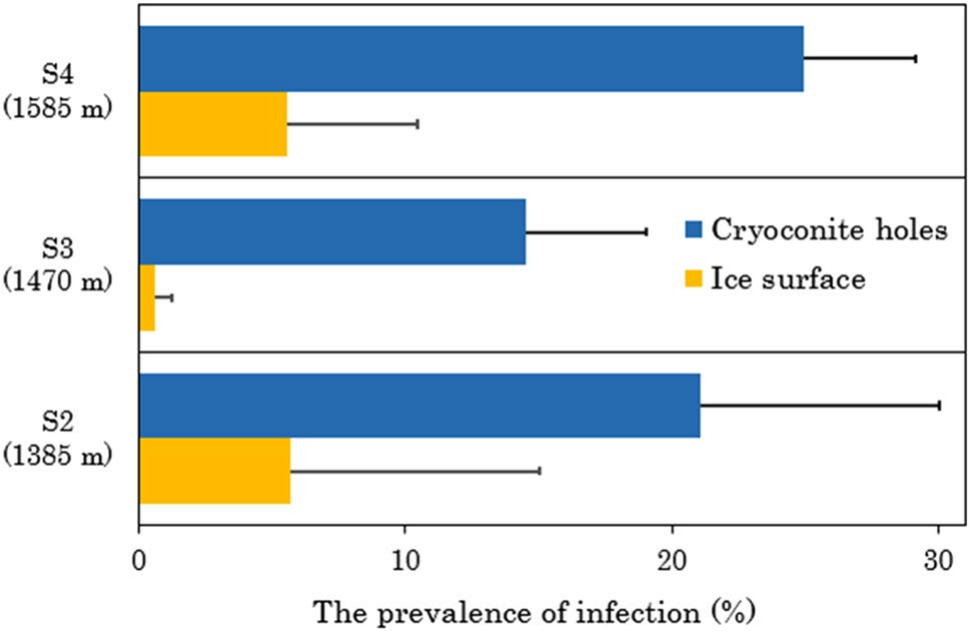
Prevalence of chytrid infections in *Ancylonema nordenskioeldii* in two different habitats (cryoconite holes and ice surfaces) at three study sites on the Gulkana Glacier. Error bars indicate the standard deviation among the four or five samples, with more than 70 algal cells counted in each sample.

The mean prevalence of infection was 5.7%, 0.6%, and 5.6% for the ice surface and 21.1%, 14.5%, and 24.9% for the cryoconite holes at the sites S2, S3, and S4, respectively (Fig. 5, Supplementary Table S2). It did not vary significantly among the study sites at different elevations (Supplementary Table S4) but varied significantly between the two habitats (the ice surface and cryoconite holes), where it was significantly higher in cryoconite holes (20.2 ± 7.3%) than on ice surfaces (3.9 ± 5.6%) for all samples (P < 0.001), regardless of elevation (Supplementary Table S3). No significant interactions between habitats and sites were detected (P > 0.05, Supplementary Table S3).

### Filament length of glacier algae

**Figure 6:**
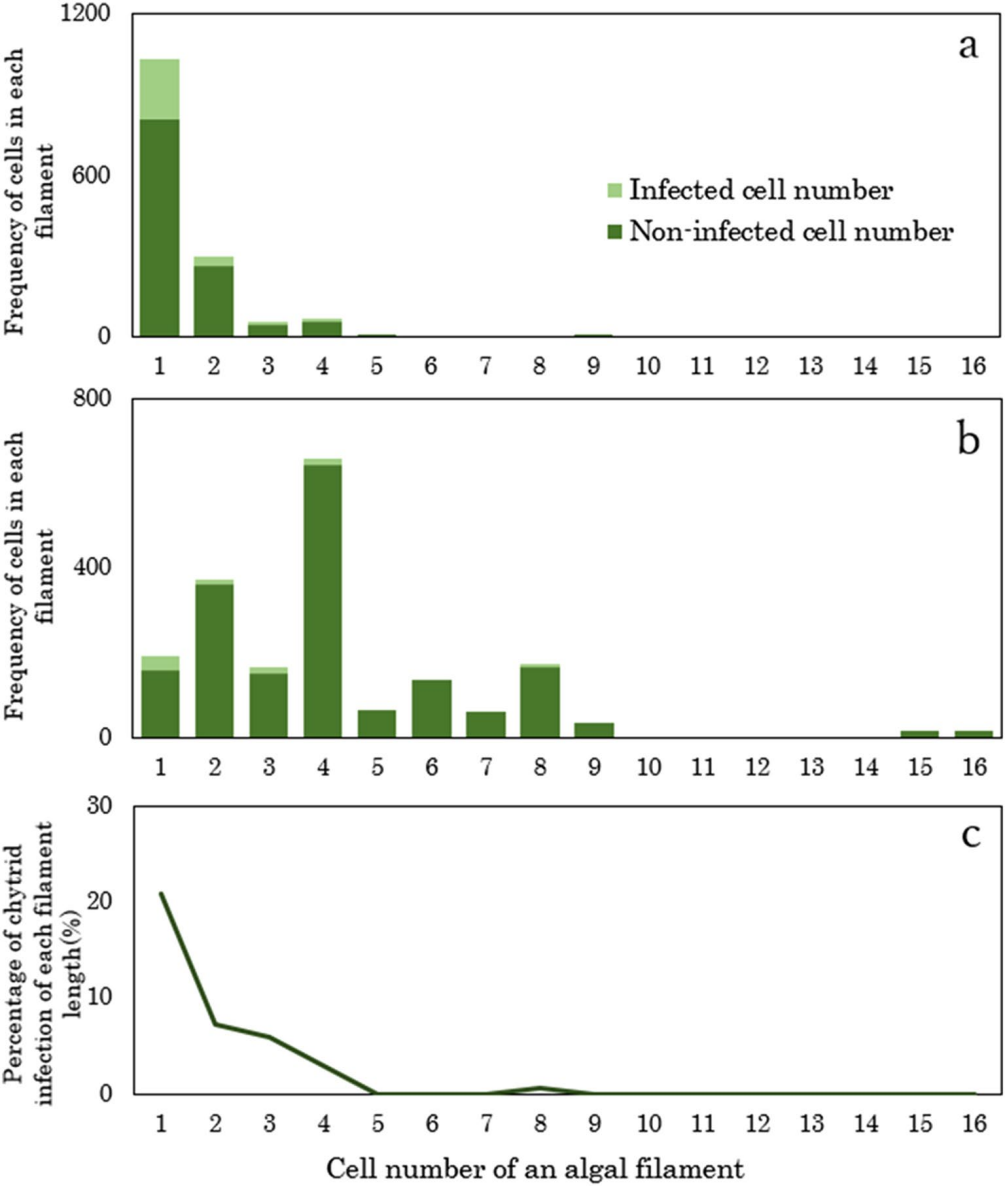
Frequency of cells in each filament with different cell numbers of *Ancylonema nordenskioeldii*. in cryoconite holes (**a**) and on ice surfaces (**b**), and the percentage of chytrid infections in each filament length (**c**). Deep and pale green bars show the numbers of chytrid-infected and non-infected cells, respectively.

As *A. nordenskioeldii* was found forming filaments consisting of 1–16 cells, we investigated whether chytrid infection varied with different filament lengths. In cryoconite holes, most algal cells (68.6%) were single (Fig. 6a). However, on the ice surface, most filaments had four cells, accounting for 35.2% of the total cells (Fig. 6b). Algae with a single cell filament showed the highest prevalence of chytrid infection, which was 20.9% of all single cells in both habitats (Fig. 6c).

## Discussion

This study showed that different morphological types of chytrids infected the glacier alga *A. nordenskioeldii* on the glacier. According to light microscopic observations of sporangia and rhizoid organization, the observed chytrids belonged to at least two genera: *Rhizophidium* and *Phlyctochytrium*. Type A chytrids likely belonged to the genus *Rhizophidium* as they were monocentric and lacked an operculum, with round sporangia outside the host cell and rhizoids observed inside the cell^44^. These morphologies were similar to those of the chytrid, *Rhizophydium sphaerocarpum*, observed previously at a glacier in Alaska^19^; however, *R. sphaerocarpum* differs slightly from Type A chytrids in that it has branched rhizoids. Type B chytrids likely belonged to the genus *Phlyctochytrium*, which is characterized by the absence of an operculum, single or multiple sporangia outside the host cell, and a single rhizoid with an apophysis extending inside the cell^45^. Type B chytrids can be distinguished from Type A ones by the presence of the apophysis. However, it was difficult to distinguish between these two types in the case of immature sporangia without a rhizoid. The identity of Type C chytrids was uncertain, as their morphology was not identical to that previously reported. As these morphological variations may also be the result of different developmental stages, single-cell molecular analysis will be necessary to determine the phylogenetic positions of these chytrids. However, the presence of sporangia of different sizes suggests the active propagation of chytrids on the glacier.

The prevalence of infection differed significantly between the two habitats but not across different elevations, suggesting that cryoconite holes could be hotspots for chytrid infections regardless of elevation. Different hydrological conditions between habitats may have affected the infectivity of the chytrids. Meltwater within the cryoconite hole is relatively static compared with that in other habitats on the glacier surface^46^. As the zoospores of chytrids swim in the water to find hosts, chytrid infections were promoted under non-turbulent conditions in a laboratory experiment^37,47^. Thus, the static meltwater in the holes may be supporting the infection of algal cells. In contrast, meltwater continuously flows on the ice surface and in gaps of the weathering crust in bare-ice areas of glaciers during the melting season^29^. Under such hydrological conditions, chytrid zoospores can be washed downstream by the meltwater flow, thus lowering the chances of algal cell infection. Therefore, the high chytrid infectivity in cryoconite holes is likely because of the static hydrological conditions in the holes.

The difference in filament length of the glacier algae across the habitats suggests that the growth rates of the host algae differs. Longer filaments were observed on the ice surface than in the cryoconite holes, indicating that the algae grew more actively on the ice surface. In contrast, single-celled stages of algae were dominant in the cryoconite holes, implying that these cells rarely divide there. As reported for many glaciers, glacier algae including *A. nordenskioeldii* and *A. alaskana* grow actively in the gaps of the weathering crust of ice surfaces^48^, probably because of better growing conditions for algae in terms of water, light, and nutrient availability. The algal cells found in the holes may have originated from the ice surface and were transferred into the holes with the running meltwater. Cryoconite holes are likely to be a suboptimal environment for glacier algae, making them more susceptible to chytrid infection. Although there are several potential explanations for the higher prevalence of chytrid infections in the cryoconite holes, the most likely is that the static water in the cryoconite holes is more suitable for chytrids and unsuitable for glacier algae, enhancing chytrid infections in the holes.

As cryoconite holes are hot spots for chytrid infections, changes in hole dynamics may largely affect the prevalence and spread of chytrids on the glacier. The dynamics of cryoconite holes have been studied by monitoring the hole size and energy balance on the glacier surface^38^. Climate change is likely to lead to changes in the number and stability of cryoconite holes, which may affect the balance between algal growth and chytrid infections.

In this study, it was revealed that a significant number of glacier algal cells were infected by chytrids. Therefore, their infection could affect the population dynamics of *A. nordenskioeldii* on the glacier surface and ultimately affect the ice melt rate, as *A. nordenskioeldii* is a key species that darkens the ice surface and accelerates its melt rate. Higher chytrid infectivity to glacier algae will increase alga mortality and thus reduce the darkening of the glacier surface. Further studies on chytrid infection dynamics are necessary to predict their role in albedo change and thus glacier melting under climate change.

## Methods

### Study site and sample collection

Samples were collected from the Gulkana Glacier in Alaska, USA (Fig. 1a). This glacier is located on the southern slope of the Alaska Range. The elevation of the glacier ranged from 1160 to 2470 m. The total area of the glacier was 16.0 km^2^ (as of 2016)^49^. The mass balance of this glacier has been monitored since the 1960s by the U.S. Geological Survey as a benchmark glacier^50^. We selected this glacier because several microbial studies have been conducted in the region^1,25,39,40^.

Sample collection was carried out on the glacier from August 4 to 6, 2015. In this study, samples were collected from three sites at different elevations (Sites S2, S3, and S4 at 1385 m, 1470 m, and 1585 m, respectively; Fig. 1d) in the bare ice area, where *A. nordenskioeldii* dominates. The weathering crust developed on bare-ice surfaces at the study sites and was covered with dispersed cryoconite or pigmented glacier algae. Five samples of the ice surface (Fig. 1c) were collected from each site using a stainless-steel scoop (1–2 cm in depth) and placed in plastic bags (WHIRL-PAK, Nasco, USA). These were melted and preserved in 30 mL plastic bottles. The cryoconite holes were ubiquitously distributed throughout the area, including the three sites. The size of the cryoconite holes (mean ± standard deviation) was 7.2 ± 3.9 cm in diameter, 8.2 ± 2.0 cm in depth, and 5.0 ± 1.9 cm in water depth. At each site, cryoconite samples from five different holes (Fig. 1b) were collected from the bottom of the holes using a pipette and were preserved in 30 mL plastic bottles. Formalin solution (3%, 1 mL) was added to all samples to fix the biological activity in the samples.

### Microscopic observation

The morphological characteristics of the chytrids parasitizing the glacier algal cells were observed using a fluorescence microscope (BX51, Olympus, Tokyo, Japan) at a magnification of × 400. First, 10–30 μL of the sample water was placed on a glass slide. One drop of 0.05% Calcofluor White and one drop of 10% KOH were then added to the sample on the slide to stain the chytrids^20,51^. The slide glass was left for at least one minute before microscopy. The glacier algae and chytrids observed in the samples were photographed using a digital camera (DP21, Olympus, Tokyo, Japan) attached to the microscope.

The cell sizes of the observed glacier algae and chytrids were measured using photographs captured with a microscope. The predominant length axis of the algal cells and the diameter of the sporangia of the chytrids were measured using image processing software (Image J 1.38X, National Institutes of Health, USA). To determine the prevalence of chytrid infection in the algal cells, the number of algal cells with and without chytrid infection was counted. More than 70 algal cells were counted in each sample. The prevalence of infection was calculated as the number of infected algal cells divided by the total number of algal cells counted.

Cells of *A. nordenskioeldii* are often connected to each other and formed filaments. To analyze the relationship between the prevalence of infection and the filament length of the glacier algae, the cell number of each filament of the algae was counted, and the frequency and chytrid infection rate of each filament were obtained for all samples.

### Statistics

The difference in the prevalence of infection among the sites (S2, S3, and S4) and between habitats (cryoconite holes and ice surfaces) and interactions between sites and habitats were tested using a two-way ANOVA followed by a post-hoc test (Supplementary Tables S3, S4). Statistical analyses were performed using RStudio software (version 4.1.2). The level of significance used in this study was 0.05.

## Supplementary Information

Supplementary Information.

## Data Availability

The datasets analyzed during this study are available from the corresponding author upon reasonable request. Correspondence and requests for materials should be addressed to K.K.
