# Supplementary material for: High prevalence of parasitic chytrids infection of glacier algae in cryoconite holes in Alaska
Source: Sci Rep. 2023 Mar 9;13:3973. doi: 10.1038/s41598-023-30721-w (PMC9998860; doi:10.1038/s41598-023-30721-w)
Supplement: Supplementary file 1 — Supplementary Information. [file 41598_2023_30721_MOESM1_ESM.pdf]

Supplementary Information

High prevalence of parasitic chytrids infection of glacier algae in cryoconite holes in Alaska

Authors: Kino Kobayashi[1]*, Nozomu Takeuchi[2], Maiko Kagami[3]

[1]Graduate school of Science and Engineering, Chiba University, Chiba, Japan.

[2]Graduate School of Science, Chiba University, Chiba, Japan.

[3]Faculty of Environment and Information Sciences, Yokohama National University, Kanagawa, Japan.

Supplementary Fig S1. Size distribution of sporangia of the chytrids observed in this study (n = 379).

Supplementary Table S1. Sizes of chytrid's sporangia of each chytrid type between two habitats (cryoconite holes and ice surfaces).

Supplementary Table S2. Prevalence of chytrid infection data from study sites (S2, S3, and S4) and habitats (cryoconite holes and ice surfaces).

Supplementary Table S3. Two-way ANOVA for the prevalence of infection between sites and between habitats.

Supplementary Table S4. Post-hoc test for the prevalence of infection between sites.

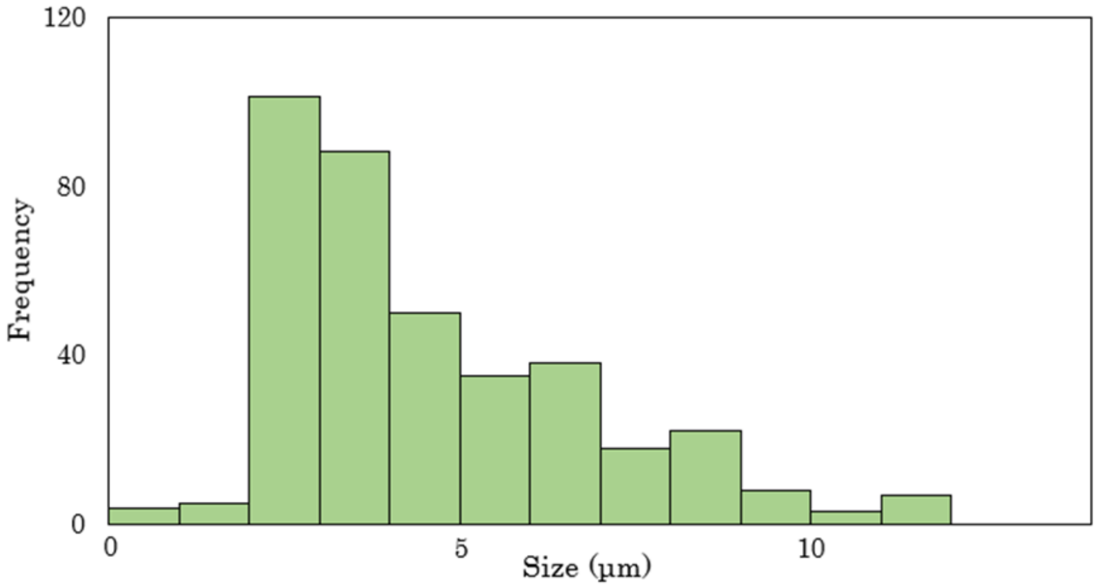

Supplementary Fig S1. Size distribution of sporangia of the chytrids observed in this study (n = 379).

Supplementary Table S1. Sizes of chytrid's sporangia of each chytrid type between two habitats (cryoconite holes and ice surfaces).

Types of chytrids	Habitat	Number of chytrid's sporangia	Size (μm)	
			Range (min–max)	Mean
A	cryoconite hole	110	1.6–11.8	3.2
	ice surface	4	1.9–3.2	2.4
B	cryoconite hole	100	2.3–11.9	6.0
	ice surface	61	2.5–11.0	5.6
C	cryoconite hole	11	2.3–5.3	3.4
	ice surface	2	3.3–3.5	3.4
Unclassified	cryoconite hole	75	0.98–11.9	4.3
	ice surface	16	2.3–9.2	4.2

Supplementary Table S2. Prevalence of chytrid infection data from study sites (S2, S3, and S4) and habitats (cryoconite holes and ice surfaces).

Site	No	Habitat	Number of non-infected cells	Number of infected cells	Prevalence of chytrid infection (%)
S2	1	cryoconite hole	60	27	31.0
S2	2	cryoconite hole	89	17	16.0
S2	3	cryoconite hole	93	18	16.2
S2	4	cryoconite hole	68	9	11.7
S2	5	cryoconite hole	71	31	30.4
S3	1	cryoconite hole	93	11	10.6
S3	2	cryoconite hole	94	14	13.0
S3	3	cryoconite hole	207	24	10.4
S3	4	cryoconite hole	112	27	19.4
S3	5	cryoconite hole	109	26	19.3
S4	1	cryoconite hole	83	23	21.7
S4	2	cryoconite hole	77	25	24.5
S4	3	cryoconite hole	81	26	24.3
S4	4	cryoconite hole	72	34	32.1
S4	5	cryoconite hole	81	23	22.1
S2	1	ice surface	144	0	0
S2	2	ice surface	102	25	19.7
S2	3	ice surface	(22)	(1)	
S2	4	ice surface	168	3	1.8
S2	5	ice surface	137	2	1.4
S3	1	ice surface	128	2	1.5
S3	2	ice surface	123	0	0
S3	3	ice surface	131	1	0.8
S3	4	ice surface	138	1	0.7
S3	5	ice surface	151	0	0
S4	1	ice surface	122	13	9.6
S4	2	ice surface	126	0	0
S4	3	ice surface	116	9	7.2
S4	4	ice surface	122	14	10.3
S4	5	ice surface	147	1	0.7

Supplementary Table S3. Two-way ANOVA for the prevalence of infection between sites and between habitats.

	df	Sum Sq	Mean Sq	F	p
site	2	344.8	172.4	4.752	**0.0187**
habitat	1	1907.7	1907.7	52.584	**2.22e-07**
site : habitat	2	39.7	19.8	0.547	0.5860
residuals	23	834.4	36.3		

Supplementary Table S4. Post-hoc test for the prevalence of infection between sites.

	diff	lwr	upr	p adj
S3-S2	-6.674444	-18.484056	5.135167	0.3533662
S4-S2	1.005556	-10.804056	12.815167	0.9756461
S4-S3	7.680000	-3.814632	19.174632	0.2393404
